# IMPContact: An Interhelical Residue Contact Prediction Method

**DOI:** 10.1155/2020/4569037

**Published:** 2020-03-25

**Authors:** Chao Fang, Yajie Jia, Lihong Hu, Yinghua Lu, Han Wang

**Affiliations:** ^1^School of Information Science and Technology, Northeast Normal University, Changchun 130117, China; ^2^Institute of Computational Biology, Northeast Normal University, Changchun 130117, China; ^3^Department of Computer Science, College of Humanities & Sciences of Northeast Normal University, Changchun 130117, China

## Abstract

As an important category of proteins, alpha-helix transmembrane proteins (*α*TMPs) play an important role in various biological activities. Because the solved αTMP structures are inadequate, predicting the residue contacts among the transmembrane segments of an *α*TMP exhibits the basis of protein fold, which can be used to further discover more protein functions. A few efforts have been devoted to predict the interhelical residue contact using machine learning methods based on the prior knowledge of transmembrane protein structure. However, it is still a challenge to improve the prediction accuracy, while the deep learning method provides an opportunity to utilize the structural knowledge in a different insight. For this purpose, we proposed a novel *α*TMP residue-residue contact prediction method IMPContact, in which a convolutional neural network (CNN) was applied to recognize those interhelical contacts in a TMP using its specific structural features. There were four sequence-based TMP-specific features selected to descript a pair of residues, namely, evolutionary covariation, predicted topology structure, residue relative position, and evolutionary conservation. An up-to-date dataset was used to train and test the IMPContact; our method achieved better performance compared to peer methods. In the case studies, IHRCs in the regular transmembrane helixes were better predicted than in the irregular ones.

## 1. Introduction

Alpha-helical transmembrane protein (*α*TMP) is an important type of membrane protein (MP) widely existing in eukaryotic cells and carrying on the responsibility of transferring signals or small molecules between two sides of biological membranes. For this reason, *α*TMPs are involved in many vital biological processes [1], such as solute and ion transport, energy transduction in respiratory and photosynthetic systems, or sensory stimuli transduction and information processing [2]. Consequently, they are major drug targets accounting for approximately 70% of the known and tested drug targets [3]. Therefore, the study of *α*TMPs' structure and function is currently a popular topic in chemistry and biology fields [4].

However, due to the specificity of the function of membrane proteins, many efforts had been applied to derive their structures, but both biological experiment and prediction approach cannot satisfy the requirements in the balance of quantity and quality. In recent years, semistructural research of TMP became more practical instead of the entire 3D structure, which is based on computational prediction to make the balance, such as hydrophobicity, electrical polarity, or contact prediction. In an *α*TMP, interhelical residue contacts (IHRCs) bind its alpha-helixes as anchor points, while most *α*TMP family members have similar transmembrane structures, so that they possibly have a similar function. It is obvious that predicting IHRCs has a special meaning for *α*TMPs compared to that of solvent proteins, although those residue contacts are totally the same in view of the biochemical background.

For the solvent proteins, considerable improvements had been made to predict the residue contact in decades, corresponding to prediction methods mainly using correlated mutation analysis- (CMA-) based or machine learning- (ML-) based methods. The CMA-based methods, such as PSICOV [5] and CCMpred [6], take into consideration that fact the contacts mostly happened between those coevolutionary residues because residue contact is an important structural feature that remains between a pair of residues on a protein sequence in the evolution process. Statistical models were mainly used in CMA-based methods to find the coevolutionary relationships from multiple sequence alignment. ML-based methods, such as CMAPpro [7], identify the contacts by abstracting various structural features of a protein sequence to a classifier. It is apparent that the coevolutionary residues can be used in an ML-based predictor to improve the prediction accuracy, which was proved by R2C [8].

The residue contact prediction still remains an open problem in the field of structural bioinformatics [5], and the above methods cannot accurately identify the residue contacts in an *α*TMP. Therefore, many TMP-specific predictors have been raised in recent decades, which are still following a similar way like those methods used on solvent proteins. After the coevolving residues were noticed relevant to the residue contact in MPs [9], the CMA-based method was applied to MPs, namely, direct-coupling analysis (DCA). Although corresponding methods continuously improved the prediction accuracy, such as mfDCA [10], PSICOV [5], plmDCA [11], and GREMLIN [12], a pure CMA-based method still has its intrinsical limitation. A direct cause is that multiple sequence alignment does not work well against the MPs, because many MPs have less family member proteins. Consequently, it is found that only a small fraction of predicted correlations involved pairs of residues in physical contact, while a sizeable fraction of the correlations was found to be in close vicinity to interhelical contacts [9]. Thus, coevolution information may not accurately detect the residue contacts by itself, but it obviously is a clue to find where the contacts exist.

For the purpose of IHRC prediction, various sequence- or structure-based features and machine learning algorithms were utilized. Neural network (NN), support vector machines (SVMs), and random forest (RF) algorithms were, respectively, proven to be effective in predicting IHRCs in TMHcon [13], TMhit [14], MEMPACK [15], TMhhcp [4], COMSAT [16], and MemConP [17]. It is convincing that the coevolution relationship highly improved the prediction accuracy used in TMHcon [13], MEMPACK [15], and MemConP [17]. These methods had already tried the best to maximize the coevolution and machine learning algorithms, but it is still a challenging problem to improve the prediction accuracy to an acceptable level, where the key factor is how to find more IHRC-specific structural features for a proper machine learning algorithm. Our previous work on interbarrel residue contact prediction for outer membrane proteins accessed a high performance method by using MP-specific features [18]. It is possible to further improve the IHRC prediction accuracy when the IHRC-specific features meet the deep learning networks, as many successful cases did in the field of bioinformatics [19–22].

In this study, we abstracted four IHRC-specific features from the *α*TMP sequences, including the Evolution Conservation Feature, Evolutionary Covariation Feature, Topology Structure Feature, and Residue Relative Position Feature. Then, a CNN- (Convolutional Neural Networks-) based predictor was proposed to predict IHRC for *α*TMPs, named IMPContact. An up-to-date dataset was used to train and test the method; the results showed that deep learning can better utilize the IHRC-specific features than the SVM, RF, and NN methods and derive higher prediction accuracy than the other classical peer methods. Considering further application, the source code of IMPContact was published at https://github.com/NENUBioCompute/IMPContact.

## 2. Materials and Methods

### 2.1. Datasets

In order to improve the reliability and authenticity of the predicted results, the data were obtained from the redundant *α*TMP sequence ID from PDBTM [23], which is a comprehensive and up-to-date transmembrane protein selection extracted from the Protein Data Bank (PDB). The dataset includes 348 nonredundant sequences, where 313 sequences were used in training and validation and the rest of the 35 sequences were used for testing.

There are three ways to label the residue contact from a protein structure: (I) the minimal distance between the heavy atoms of the side chain or backbone was less than 5.5 Å [13]; (II) the sum of their van der Waals (VDW) radii plus a threshold of 0.6 Å [14] (the VDW radii were taken from Li and Nussinov in 1998) [24]; and (III) a maximal distance of 8 Å between their C-beta atoms (C-alpha for glycine) [15]. Since it is essentially the same as the three definitions, MEMPACK [15] found that the prediction accuracy will be less different regardless of what definition was selected, and we used the more restricted definition (I) to label the IHRC for an *α*TMP.

### 2.2. TMP-Specific Sequence Features

As known, distinctive features will be highly helpful for a machine learning method to make a better prediction. The IHRCs in an *α*TMP have many specific features observed in transmembrane segments. We selected four structural features to describe the existence of IHRCs from different perspectives, where an evolution conservation feature is widely used in protein research and proved to be available to describe the conservation sequence segments, a topology feature is used to describe the transmembrane segments, an evolutionary covariation feature is used to indirectly reveal the existing residue-residue contacts, and residue relative position features are used to enhance the structural information.

#### 2.2.1. Evolution Conservation Feature

Multiple sequence alignment discovered the evolution conservation against the large-scale protein sequence database; it had already been wildly applied in various biological sequence researches [18, 25]. More than 30% of the homologous superfamilies described in CATH are composed mainly or entirely of *α*-helixes [26]. Transmembrane helixes are different from those in the soluble proteins because the environment in the lipid layer force those helixes that were stretched. But the evolution conservation becomes more distinct and special in these sequence segments, leading to a different way to descript the evolutionary conservation between a pair of residues that have IHRC. At this point, evolution conservation is a particular TMP-specific feature.

The evolution conservation of a residue can be described by the PSSM (Position-Specific Scoring Matrix) produced from a multiple alignment tool; it presents the frequency of a residue type that appeared in each position of the protein sequence. The raw PSSM profile is represented by a 20-dimensional score vector [16, 27]. We obtained the PSSM by running a stand-alone PSIBLAST [28] against NCBI's nonredundant sequence database (NR) with three iterations and the *E* value set to 1*e*‐10.

#### 2.2.2. Evolutionary Covariation Feature

Different from evolution conservation, evolutionary covariation is aimed at descripting the evolutionary correlation between two residues on a protein sequence. It was observed that the residues having contact possibly present highly evolutionary conservation, because the contacts are not randomly existing in the protein, and they are closely related to the protein structures. But on the contrary, not all the residue pairs with evolutionary conservation will have contact. Therefore, conservation and covariation present the evolutionary information of a protein sequence from discriminate perspectives.

All methods generate the evolutionary covariation from the multiple sequence alignments; we allow IMPContact to accept the evolutionary covariation feature abstracted by different methods under the condition that those methods can be integrated into IMPContact, where the selected method uses the multiple alignments generated from the process we did for the evolution conservation feature then calculates the evolutionary covariation for all the possible residue pairs on a TMP. As known, different evolutionary conservative tools may have different numerical spaces when predicting different protein sequences, so the evolutionary covariation feature finally inputting to IMPContact will be standardized using the *z*-score for each sequence.

#### 2.2.3. Topology Structure Feature

As mentioned, the helixes are special in the transmembrane domain in the TMPs compared to soluble proteins; they are characterized by the topology structure to distinguish the transmembrane and nontransmembrane segments. There is no doubt that the topology structure will directly reduce the searching space for the IHRCs, and it is exactly a TMP-specific feature. According to our goal, only sequence-based features are suitable for use in the prediction and the predicted topology structure meets the requirement.

There are several methods available to predict the topological structure of intimal proteins, including some that are based on hydrophobicity analyses [29], statistical procedures [30], or machine learning-based methods [31, 32]. The accuracy is the most important factor in choosing a topology structure prediction method; in our previous work, DMCTOP [33] was used to abstract the topology structure features. Another consideration is that the DMCTOP is a deep learning-based method, and it was upgraded during the further improvement of IMPContact.

The predicted topological structure was output in a uniform format, in which the cross-diaphragm residues were identified by the character “H”, the outer residues by the character “o/O”, and the inner residues by the character “i/I”. Eventually, all the characters form a sequence equal to the length of the original protein sequence. The predicted topology structures were digitized into a vector as one input feature.

#### 2.2.4. Residue Relative Position Feature

Relative position is a derivative feature from the topology feature. According to the observation, the transmembrane helixes on a sequence always alternately cross the membrane, resulting in IHRCs not occurring between the residues that separately close to the different sides of the membrane. Here, the relative position feature was used to descript whether the two residues on the neighboring helixes close to the same side of the membrane are close to each other.

The relevant position of a residue was assigned depending on the predicted topology structure. The process started from the N-terminal of the sequence. We assigned increasing integer values starting from 1 for each residue on each odd transmembrane helix that appeared and then did the same to the rest of the transmembrane helixes from the C-terminal; the other residues were assigned to -1 finally. After the above process, all the residues had a value representing their relative position; those residues who had closing positive values are considered more possibly closing to each other.

### 2.3. Sliding Windows

The IHRC is a local residue interaction on the TMP sequence, though it possibly happens between any pair of transmembrane helixes. The remote residues of the protein structure have fewer influences on IHRC, while the structural neighboring residues are mainly involved in forming the necessary surrounding environment. For this reason, two sliding windows with a size of 5 were used to characterize the features for both residues of a contact pair, where the 2 upstream and 2 downstream residues were included in the sliding window with the first candidate residue, and the same sliding window was applied to the other candidate residues. In each turn, the 4 TMP-specific features of the residues in the two sliding windows constituted a one-dimensional feature vector for the corresponding candidate pair of residues, in which an evolution covariation feature was used only between the two candidate residues. For the contact prediction between residues *Ai* and *Bj*, the features were abstracted from residues (*A*_*i*−2_*A*_*i*−1_*A*_*i*_*A*_*i*+1_*A*_*i*+2_) and (*B*_*j*−2_*B*_*j*−1_*B*_*j*_*B*_*j*+1_*B*_*j*+2_), where *i* and *j* are the sequence positions and *i* ≠ *j*. According to the sliding window, an SVM input *V*_*i*,*j*_ is given as follows:

(1) $$\begin{array}{c} V_{i,j}=\left(C_{i,j},{\left(E,T,R\right)}_{i-2},{\left(E,T,R\right)}_{i-1},{\left(E,T,R\right)}_{i},{\left(E,T,R\right)}_{i+1},{\left(E,T,R\right)}_{i+2},{\left(E,T,R\right)}_{j-2},{\left(E,T,R\right)}_{j-1},{\left(E,T,R\right)}_{j},{\left(E,T,R\right)}_{j+1},{\left(E,T,R\right)}_{j+2}\right), \end{array}$$

where (*E*, *T*, *R*)_*i*_ are the evolution conservation feature, topology feature, and relative position feature of residues in position *i*, respectively, and C_*i*,*j*_ is the predicted evolutionary covariation between residues *i* and *j*. Finally, a 31-dimensional eigenmatrix is formed. In this way, it became a simplified binary classification problem to predict IHRCs.

### 2.4. Deep Learning Network

As a representative branch of deep learning, CNN [34] had already made great achievements in various research fields, including protein sequence studies [35–39]. It can capture various nonlinear features by constructing neural networks consisting of convolution, pooling, and fully connected layers. The learning capability of CNN is strongly supported by diversification through the assembly of the different layers, activation functions, and ways of connecting the nodes into the networks. Therefore, lots of CNN-based applications have been raised to solve various kinds of researching issues in recent years; it has high adaptability in keeping improvements.

**Figure 1:**
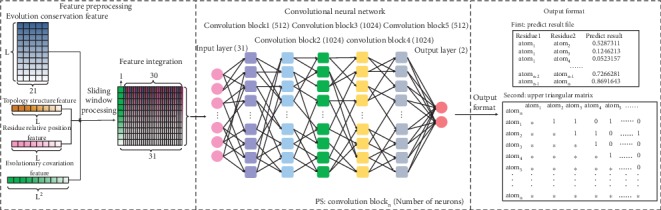
The neural network construction of IMPContact.

According to the sliding window feature, we constructed the IMPContact prediction process with a CNN-based model kernel, as shown in Figure 1. IMPContact allows users to input a whole TMP sequence; all the TMP-specific features will be generated at one time, then it scales the whole sequence from the N-terminal to the C-terminal using two sliding windows, until all the residue pairs are predicted by the CNN-based kernel. Finally, it outputs the prediction results into two text files: one is the list of residue pairs marked with a prediction conclusion and the other one is an upper triangular matrix recording the prediction conclusion accordingly.

The CNN kernel was designed as follows:

1. Input layer: the input is a 31 × 1 feature vector, corresponding to the residue features in the two sliding windows; the middle residues in the windows are the candidates to be predicted
2. Convolutional block layers: there are 5 layers of convolutional blocks—512, 1024, 512, 1024, and 1024. All the block layers are fully connected, and all the blocks use the same network, which is composed of a convolutional layer (kernel = 3), a rectified linear unit (ReLU) layer, and a max pooling layer
3. Output layer: the output is the binary classification result for each input residue pair

## 3. Results and Discussion

### 3.1. Evolutionary Covariation Method Selection

Different from the other features used in this work, the evolutionary covariation feature is the key to accurately identify the existence of IHRCs. As mentioned previously, the evolutionary covariation relationship between a pair of residues was obtained from the multiple alignments against a particular protein sequence database, but the process cannot guarantee that those residue pairs with high evolutionary conservation must have residue contact, especially for the protein sequences that have fewer homology proteins in the sequence database. This is the reason why the evolutionary covariation is an indirect feature so that choosing the method to abstract this feature becomes important.

There were three evolutionary covariation methods taking into consideration IMPContact, namely, ELSC [40], MI [41], and OMES [42]. In spite of the many similar methods providing computational tools, e.g., EVFOLD [43], which obtained an even higher accuracy on covariation, those 3 methods are more convenient to realize an easy-to-use particular IHRC method for the users. For the purpose of discovering the covariation residue pairs from the multiple alignment result, ELSC (Explicit Likelihood of Subset Covariation) uses combinatorial arguments to realize a perturbative algorithm [44]. MI (mutual information) measures the codependency of two residues as random variables [45]. OMES iteratively calculates the score for each pair of residues by the frequency they observed in the sequence alignment based on a correlated analysis method [46].

The above three evolutionary covariation methods were discussed having different performances on different datasets [42], and we tested all the methods in our method to choose the best one with the highest compatibility to the IMP alignment results, where some TMPs have rare homology protein sequences, and IHRC has special distribution of the evolutionary covariation relative to the soluble proteins caused by standing in the lipid layer environment. In comparison, the evolutionary covariation features were abstracted using the ELSC, MI, and OMES methods and, respectively, input into the IMPContact while the other features and the deep learning networks are completely the same. The experiments randomly selected 80% of the sequences from the training dataset to train the prediction models corresponding to the three covariation methods then used the other 20% of the sequences to test the three models.

As shown in Table 1, the three methods obtained obvious prediction performances. ELSC provided the highest accuracy both on ACC and MCC. MI obtained the highest ACC of 0.9742 but failed to get an acceptable MCC; it is obvious that this method could not help the IMPContact to distinguish the positive and negative samples, because most residue pairs in the transmembrane helixes were considered having a similar covariation, and it ignored those residue pairs with contact that should have a stronger covariation. Compared to the other two methods, the OMES obtained a middle accuracy on both standards. Therefore, the ELSC is more compatible with our model to descript the evolutionary covariation feature. All the following tests and publishing models used the ELSC method.

### 3.2. Classifier Comparison

Deep learning methods were proven to be inefficient for all the studies. They depend on many factors, such as data space, data distribution, and the researching problems; there is no guarantee that a deep learning method could be better at solving the IHRC prediction compared to traditional machine learning methods. Therefore, we did the prediction performance comparison among our CNN-based model and three widely used machine learning methods: SVM (Support Vector Machine) [47–49], RF (Random Forest) [50, 51], and NN (Neural Network) [52, 53].

The CNN model was built on the PyTorch platform [54]; the other three machine learning methods used the Scikit-learn toolkit [55]. All the models were developed using the Python language and trained and tested against part of our training datasets the same as that in Section 3.1. The parameters of each model were optimized to obtain the best prediction accuracy correspondingly. The same four TMP-specific features were input to each model for all the TMP sequences; among them, the evolutionary covariation feature was abstracted using the ELSC method, which was proven to be the most compatible to the IHRC prediction.

The prediction accuracy levels of the four methods are listed in Table 2; the CNN model achieved the best performance with the highest MCC and ACC, which were about 0.34 and 0.84, respectively. The other three machine learning methods obtained obviously lower accuracy levels on both criteria, where the RF model had the worst accuracy with an MCC of about 0.12 and an ACC of about 0.63; the SVM and NN models were better than the RF model with a little higher accuracy. Although the NN model obtained a closer ACC to the CNN model, and a better MCC value, none of the machine learning methods had accessed an acceptable MCC as that of the CNN model. The results showed that those three methods predicted more negative samples to be positive samples, casing the lower MCCs even when high ACCs appeared, not to mention that the RF and SVM models had much lower ACC values. Finally, the CNN model was chosen as a classifier used in the IMPContact.

### 3.3. Prediction Performance

#### 3.3.1. Cross-Validation on the Training Dataset

Fivefold cross-validation was used for our method on our training dataset to show the training performance. In this experiment, all the 4 IMP-specific features and the prediction classifier had already been chosen according to the above processes. All the training sequences were randomly assigned to 5 subsets with similar members; in each fold, one subset was used as the testing dataset, while the other four subsets were used as the training datasets. The validation process was completed when each subset had been tested; there were a total of 5 models produced during the training and testing processes. At the same time, traditional machine learning methods were validated using the same steps to comprehensively represent that the deep learning method performed better than the other methods in this issue. Here, the MCC was used to evaluate the performance as a balanced criterion.

As known, fivefold is not an extremely strict cross-validation; it was adopted in this experiment determined mainly by the size of the TMP dataset. In our nonredundant training dataset, many TMPs have only a few homology proteins to obtain the comprehensive evolutionary investigation, which will mislead the testing when too many folds are used in the cross-validation. Moreover, fewer folds cannot descript the stability of the model.

**Figure 2:**
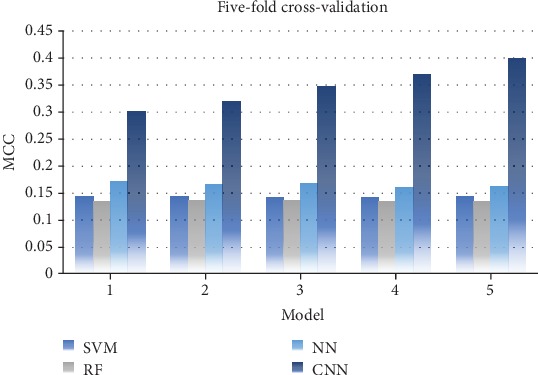
Prediction model comparison on fivefold cross-validation.

The prediction accuracy is shown in Figure 2; the CNN-based model obviously surpasses the other three machine learning methods, while they had a similar performance. It can be found that the IMPContact could obtain a consistent performance in the cross-validation no matter what classifier was taken, where all the models were stable in the prediction. The phenomenon illustrates that TMP-specific features had strongly supported the prediction, but they had no such clear data space bundle for the traditional machine learning methods to distinguish the IHRCs, while the CNN model found more details by deep learning.

#### 3.3.2. Sequence Length Distribution in the Datasets

In previous studies, TMPs' protein family was found strongly relative to their sequence length; this is because the TMPs are mostly remotely homological to have the conservative transmembrane segments and the functional segments. Therefore, the distributions of the training dataset and testing dataset should be discussed before the final testing.

**Figure 3:**
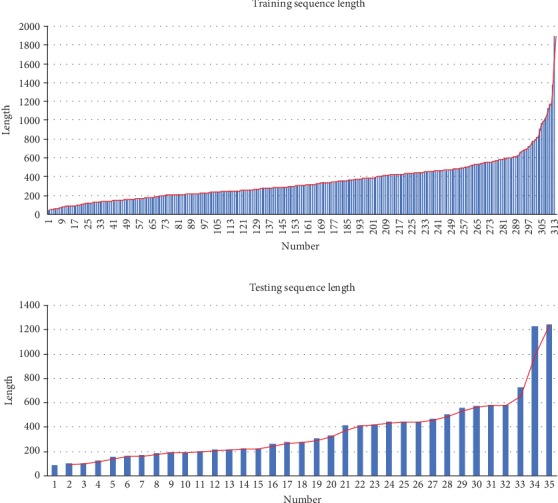
Sequence length distributions of datasets.

We counted the sequence lengths in both the training and testing datasets, as shown in Figures 3(a) and 3(b). The diagram in Figure 3(a)is the sequence length distribution of all the training datasets, and the one in Figure 3(b) represents that of the testing dataset. It can be found that both datasets have a similar distribution in sequence length descripted using the red lines, where most sequences are less than 600 amino acids, a few sequences are 600-800 amino acids in length, and a small number of sequences are larger than 800 amino acids. It is obvious that our testing results would be more accurate under the condition that the training and testing datasets have such similar distribution. There is another problem that those long sequences have more complex IHRCs than short sequences, and they may not obtain enough training. However, IMPContact used a sliding window of 5 residues to descript the features of the candidate contact pairs and it will be less affected by the sequence length as the local features.

#### 3.3.3. Prediction Performance

By the above studies, the final IMPContact was built using a CNN-based deep learning model; accepting the 4 TMP-specific features as inputs, the ELSC method was chosen to abstract the evolutionary covariation feature, outputting the IHRC prediction results into two formats. Here, we excluded the peer methods which are unavailable or proved to have low accuracy, and the methods were not suitable for IHRC prediction; thus, PSICOV and CCMpred were selected to make the comparison. Both the two methods are CMA-based methods, but they did not use any TMP-specific features and machine learning models. We downloaded the stand-alone tools of both methods to input the multiple alignment results the same as that of the IMPContact. The experiment used our testing sequences. To comprehensively descript the prediction performance, ACC, Precision, Recall, and MCC evaluation criteria were, respectively, counted using the prediction results.

As shown in Table 3, a total of 35 testing sequences were input into the three methods. PSICOV obtained the prediction results for 13 sequences, while the other two methods were available for all the sequences, and the performances were calculated based on those predicted samples by each method. Additionally, precision was used instead of ACC in a few peer studies, so we list both values. CCMpred had not predicted any residue pairs having contact, and the negative samples were far more than the positive samples, so it obtained a high ACC, while Precision, Recall, and MCC could not be calculated. PSICOV was better than CCMpred, but still conservative in positive sample prediction; consequently, it obtained a high ACC, while the other evaluation criteria were low. In contrast, IMPContact achieved the best performance; although there were more false positive samples predicted causing a lower ACC, it had a much higher MCC than PSICOV.

The comparison above revealed that the evolutionary covariation analyses cannot efficiently predict the IHRC currently, because less homological protein sequences limited the multiple alignments to discover the evolutionary information for TMPs, both in conservation and covariation. TMP-specific features are necessary to improve the prediction by bringing in more structural features to the classifier. Meanwhile, deep learning can better capture those TMP-specific features to identify the IHRCs.

### 3.4. Case Studies

**Figure 4:**
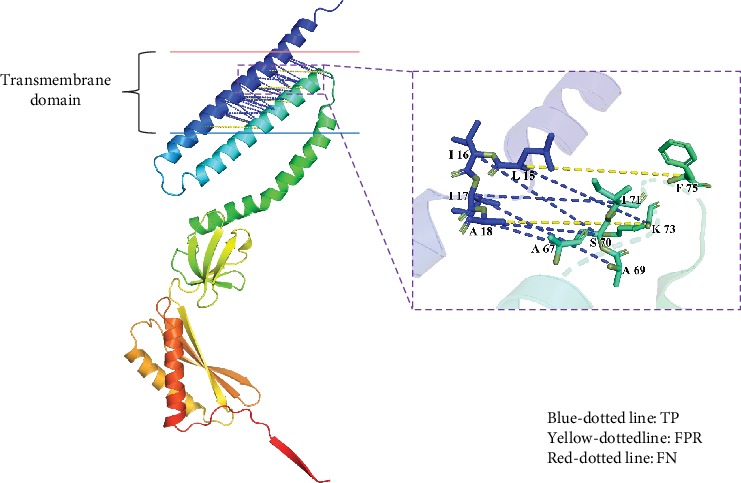
Prediction case of 3UDC_A.

**Figure 5:**
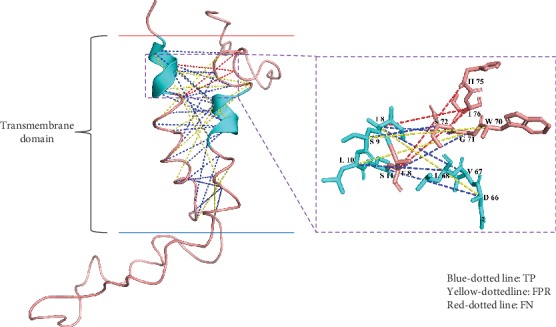
Prediction case of 2WSC_G.

For the purpose of displaying the details of the prediction, 3UDC_A [56] and 2WSC_G [57] were chosen as representative causes shown in Figures 4 and 5, where the residues in a true positive sample were connected using a blue dotted line, as well as yellow and red dotted lines for the false positive and false negative samples, respectively.

The 3UDC_A belongs to a mechanosensitive channel, which is composed of seven domains having the same sequences. Each domain has two transmembrane helixes along with one helix half crossing the membrane. 3UDC_A is a classical TMP, with two transmembrane helixes binding together through IHRCs. In this case, all positive contacts were correctly predicted, a few false positives were made close to the positive ones, and no false negative was predicted. It illustrated that the IMPContact is efficient for predicting the IHRCs between the regular transmembrane helixes.

In the other case, the 2WSC_G comes from a huge protein complex Plant Photosystem I. The transmembrane segments are partly formed as a helix; the rest of the parts mostly are flexible structures. The 2WSC_G has no such regular transmembrane helixes as in 3UDC_A; it directly weakened all the features helping IMPContact to make the classification, not only the evolutionary relevant features but also the topology relevant features, so that much more false positives and even false negatives appeared. It is extremely hard for our method to accurately predict the IHRCs for an irregular TMP, especially when the transmembrane segments are affected by the other segments in the protein complex.

## 4. Conclusions

In this study, we proposed an IHRC prediction method for inner transmembrane proteins. The TMP-specific features were used as inputs representing evolutionary and topology structure information, and a CNN model was used as the classifier. After the experiments, the ELSC method proved to be better at discovering the evolutionary covariation in transmembrane segments, but it cannot identify the IHRCs by itself. The deep learning method showed that it was efficient in predicting IHRC based on the TMP-specific features. Compared to the CMA-based methods, our method achieved better performance on a testing dataset. In the case studies, IHRCs in the regular transmembrane helixes were better predicted than in the irregular ones. It is still a challenge to accurately predict IHRCs for all the TMPs.

## Figures and Tables

**Table 1:** Comparison of candidate evolutionary covariation methods.

Methods	ACC	MCC
ELSC	0.8408	0.3371
MI	0.9742	0
OMES	0.7122	0.1335

**Table 2:** The prediction accuracy comparison of 4 classifiers.

Methods	ACC	MCC
RF	0.6337	0.1209
SVM	0.7451	0.1478
NN	0.8050	0.1745
CNN	0.8408	0.3371

**Table 3:** Comparison with peer methods.

Methods	ACC	Precision	Recall	MCC	Predicted samples
CCMpred	0.9978	—	—	—	35
PSICOV	0.9878	0.0030	0.3842	0.0166	13
IMPContact	0.6293	0.0035	0.5920	0.0271	35

## Data Availability

All the Training and Testing datasets are available at https://github.com/NENUBioCompute/IMPContact.
